# Complete mitochondrial genomes confirm the generic placement of the plateau vole, *Neodon fuscus*

**DOI:** 10.1042/BSR20182349

**Published:** 2019-08-09

**Authors:** Jian-Qiu Li, Li Li, Bao-Quan Fu, Hong-Bin Yan, Wan-Zhong Jia

**Affiliations:** 1 State Key Laboratory of Veterinary Etiological Biology/ National Professional Laboratory of Animal Hydatidosis/ Key Laboratory of Veterinary Parasitology of Gansu Province/ Lanzhou Veterinary Research Institute, CAAS, Lanzhou 730046, P.R. China; 2 Jiangsu Co-innovation Center for Prevention and Control of Important Animal Infectious Disease, Yangzhou 225009, P.R. China

**Keywords:** Arviciolinae, Mitochondrial genome, Neodon fuscus, Phylogenetic analysis, Taxonomy

## Abstract

The plateau vole, *Neodon fuscus* is endemic to China and is distributed mainly in Qinghai Province. It is of public health interest, as it is, a potential reservoir of *Toxoplasma gondii* and the intermediate host of *Echinococcus multilocularis*. However, genetic data of this species are lacking, and its name and taxonomy are still a controversy. In the present study, we determined the nucleotide sequence of the entire mitochondrial (mt) genome of *N. fuscus* and analyzed its evolutionary relationship. The mitogenome was 16328 bp in length and contained 13 protein-coding genes, 22 genes for transfer RNAs (tRNA), two ribosomal RNA genes and two major noncoding regions (O_L_ region and D-loop region). Most genes were located on the heavy strand. All tRNA genes had typical cloverleaf structures except for tRNA^Ser (GCU)^. The mt genome of *N. fuscus* was rich in A+T (58.45%). Maximum likelihood (ML) and Bayesian methods yielded phylogenetic trees from 33 mt genomes of Arvicolinae, in which *N. fuscus* formed a sister group with *Neodon irene* and *Neodon sikimensis* to the exclusion of species of *Microtus* and other members of the Arvicolinae. Further phylogenetic analyses (ML only) based on the *cyt*b gene sequences also demonstrated that *N. fuscus* had a close relationship with *N. irene*. The complete mitochondrial genome was successfully assembled and annotated, providing the necessary information for the phylogenetic analyses. Although the name *Lasiopodomys fuscus* was used in the book ‘Wilson & Reeder’s Mammal Species of the World’, we have confirmed here that its appropriate name is *N. fuscus* through an analysis of the evolutionary relationships.

## Background

Mitochondria (mt) are virtually ubiquitous in eukaryotic organisms, playing an important role in a range of cellular processes. With few exceptions, metazoan mt genomes are covalently closed-circular molecules. In the case of mammals, these mt genomes range from 15 to 17 kb. In addition to the D-loop, which is involved in initiation of DNA replication, mt genomes contain 13 protein-coding genes: cytochrome b (*cyt*b), subunits 1-3 of cytochrome oxidase (*cox*1-3), ATPase subunits 6 and 8 (*atp*6 and *atp*8) and NADH dehydrogenase subunits 1-6 and 4L (*nad*1-6, *nad*4L). Furthermore, they have genes for two ribosomal RNAs (rRNA) (12S rRNA and 16S rRNA) and 22 transfer RNAs (tRNAs). Although mt genomes are rather uniform in their length and structure, the primary sequences of their genes vary substantially. Because of their small size, simple structure, fast evolutionary rate, maternal inheritance, limited recombination and lack of tissue specificity [1–4], mtDNA sequences are used widely for analyzing phylogenetic relationships among vertebrates and for population differentiation [5–7].

The plateau (or smoky) vole (a rodent in the family Cricetidae, subfamily Arvicolinae) was first described as *Lasiopodomys fuscus* by Büchner in 1889 [8]. Its taxonomy has been unstable since then, with the species being referred to as several different subgenera/genera in recent years, in particular *Microtus* Schrank 1798, *Lasiopodomys* Lataste 1887 and *Neodon* Horsfield 1841. The authoritative ‘Wilson & Reeder’s Mammal Species of the World’ (third Edition, 2005) placed it in *Lasiopodomys* (see http://www.vertebrates.si.edu/msw/mswcfapp/msw/taxon_browser. cfm?msw_id = 4367), and this classification is commonly accepted (e.g., Smith and Xie) [9]. Liu et al (2012) [10] considered, partly on the basis of mt cytochrome *b* sequences, that the species should be transferred from *Lasiopodomys* to *Neodon*. This was also demonstrated, but not explicitly stated, by Chen et al (2012) [11] in a study using mitochondrial and nuclear DNA sequences. In the Chinese animal scientific database, the name of *L. fuscus*, however is also used, the Chinese public health literature, related to plague and other pathogens (see below), almost invariably uses the name *Microtus fuscus*.

This plateau vole is the natural host of a variety of parasites. Research has shown that the rate of *Toxoplasma gondii* infection was 12.5% (6/48) and that this species could serve as a potential reservoir of *T. gondii* [12]. Qinghai-Tibet Plateau is one of the two major Alveolar echinococcosis (AE, caused by *Echinococcus multilocularis* larvae) endemic areas (the other is Xinjiang) in China, and the vole is an endemic species and one of the endemic small mammals, and it plays a significant role in transmitting *E. multilocularis* [13]*.* According to our survey, the rate of *E. multilocularis* infection was 29.41% (32/102) in Jiuzhi county, Qinghai province [14]. Xu et al [15] found that the rate of *E. multilocularis* infection was different as the seasons changed, and that that in summer, the infection rate (3.4%, 12/353) was lower than in winter (11.66%, 76/652). Moreover, its fleas have been confirmed as infected with the natural plague (*Yersinia pestis*) [16,17]. A new study found that *Neodon fuscus* could also be the host of *Taenia tianguangfui* n. sp. [18]. It is, therefore, an actual or potential reservoir of animal and human zoonotic pathogens. Therefore, it is necessary to name this vole and to understand its taxonomy and phylogenetic relationships with other rodents to aid epidemiological investigations of zoonoses.

In the present study, we report the entire nucleotide sequence of the mt genome from the plateau vole, including information on the gene arrangement, nucleotide composition and codon usage. In addition, the phylogenetic and systematic position of this species is investigated. Based on the analysis of phylogenetic trees inferred from complete mt genomes and *cyt*b genes, we strongly suggest that the vole examined in this study should be named *N. fuscus*.

## Materials and methods

### Samples and DNA extraction

The specimens of *N. fuscus* were collected from Wow Aryi Xiang, Jiuzhi County, Golog Autonomous Prefecture, Qinghai Province (33°19′N, 100°32′E) with an average elevation of 4100 meters above sea level. The collection and autopsy of the trapped voles was strictly conducted under national ethical guidelines (Regulations for Administration of Affairs Concerning Experimental Animals, China, 1988) for animal husbandry and humane treatment. Total genomic DNA was extracted from liver tissue using the TIANamp Genomic DNA Kit (TianGen).

### PCR amplification, sequencing and identification

Primers were designed with reference to an alignment of sequences from published mt genomes of voles (Supplementary Table S1). Nine overlapping PCR products, ranging from 1829 to 2163 bp in length, were amplified from one specimen (specimen 1) to cover the entire mt genome. Primers used for the generation of PCR products and the sizes of the fragments are given in Table 1. PCR was carried out using a standard 3-step cycle: 94°C, 4 min, 35 cycles of: 94°C, 30 s, 52–56°C, 30 s, 72°C, 2 min 30 s followed by hold at 72°C, 10 min and a final hold at 16°C.

**Table 1:** Primers used to amplify the complete mitochondrial genome of *N. fuscus*

Primer name	Primer sequence (5′→3′)	Positions on the H-strand	Size of PCR product (bp)
F1	TGA AAA TGC TTA GAT GGA TGC	30–50	2123
R1	CTC CAT AGG GTC TTC TCG TC	2133–2152	
F2	GGT AGC ATA ATC ACT TGT TC	2009–2028	
R2	GCT TTA TTA GCT GAC CTT AC	3819–3838	1829
F3	CCC TAC ACC TAG AAA TAT GTC TG	3670–3692	
R3	CCC TGC TTC TAC TAT TGA TG	5642–5661	1992
F4	CTC AGC CAT TTT ACC TAT GTT C	5283–5304	
R4	TGT TTT TAC TGT GAG GGC TG	7259–7278	1995
F5	TCC AAC TAG GCT TAC AAG ATG	6995–7015	
R5	AGA YCC RTA AAT TCC GTC TG	9139–9157	2163
F6	ACG RAA CAR YAT AAA CCA AGC	9038–9058	
R6	GGA TTA TRA TAG CGG TGA TGA C	11022–11043	2006
F7	ATG RGG TAA CCA AAC AGA ACG	10538–10558	
R7	GTT GGC TTG ATG TTG AGA ATG	12612–12632	2095
F8	AAC ARY ACY ATC YTR ACA GCC	12512–12532	
R8	TGA GGG TRG CTT TRT CTA CTG	14620–14640	2129
F9	TGA GGA CAA ATA TCA TTC TG	14517–14536	
R9	ATG TAC TTG ATA CCC TCT CC	170–189	2001

Each PCR product purified for sequencing was gel-cut and DNA was recovered through a column (AxyPrep DNA Gel Extraction Kit by AxyGen). The prepared products were sent to GENEWIZ to be sequenced using Sanger dideoxy chain termination in an ABI3730 DNA Analyzer. All the raw sequences were assembled, edited and aligned using the software package SeqMan (DNASTAR7.1) [19], Clustal W [20] and Bioedit v7.2.3 [21]. The boundaries of protein-coding genes and ribosomal genes were predicted by sequence homology with those of other voles. tRNAscan-SE1.2.1 [22] was used in a preliminary search for tRNA genes using the default search mode, the vertebrate mt genetic code (transl_table = 2) and ‘Mito/chloroplast’ source.

To examine the genetic variation of mtDNAs among additional individuals, primers were also designed for amplifying only the *cyt*b gene (forward primer: 5′-CAAAGAAGTGCCTAAACAACCTA-3′; reverse primer: 5′-TAGAATATCAG CTTTGGGTGTTG-3′; expected length: 1402 bp) and D-loop region (forward primer: 5′-TCTCAGGGCATCAAGAAGGAAGG-3′; reverse primer: 5′-TGTGGCTAGGC AAGGTGTCTTTA-3′; expected length: 1231 bp) from 36 individuals from the area where specimen one was collected. PCR and sequencing of the *cyt*b gene and D-loop region was performed according to the abovementioned description.

### Phylogenetic analyses

To investigate the phylogenetic position of *N. fuscus* relative to other rodents, we used ClustalW to align the complete mt genomes of 32 rodent species which were downloaded from GenBank (Supplementary Table S2). Trees were constructed using maximum likelihood (ML) approaches (GTR+I+G4 model; 1000 bootstraps) in IQ-TREE [23] and Bayesian methods in MrBayes v3 [24]. The Bayesian analysis was run for 500000 generations and sampled every 1000 generations. The first 25% of trees were omitted as burn-in and the remaining trees were used to calculate Bayesian posterior probabilities. The mtDNA sequences of two species of pikas, *Ochotona collaris* and *Ochotona* curzoniae, were used as outgroups. Because the mt *cyt*b is often used as a molecular marker for phylogenetic studies in the Arvicolinae, we also constructed trees from *cyt*b genes of the same 34 (33 ingroup; 1 outgroup) species. The phylogenetic tree based on mt *cyt*b genes was constructed using ML algorithms implemented in online IQ-TREE Web Server (http://iqtree.cibiv.univie.ac.at/) and TIM2+F+I+G4 model was chosen according to AIC implemented in IQ-TREE. Similarly, the tree based on mt *cyt*b genes was also constructed by MrBayes v3.

## Results

### Genome structure and base composition

The complete mt genome of *N. fuscus* (specimen 1) obtained in the present study was a closed circle of 1328 bp (Accession number: MG833880/ NC_040138) (Table 2). The genome was comprised of two noncoding regions, 13 protein-coding genes, 22 tRNA genes and two rRNA genes, a structure similar to those of other rodents. The mtDNA base composition was 31.91% A, 14.36% G, 26.54% T and 27.20% C.

**Table 2:** Nucleotide composition data for the mitochondrial genomes of *Neodon fuscus* and other rodents

Family or Subfamily (Species)	Size (bp)	Nucleotide compositions of complete mt sequence				Nucleotide compositions of 13 protein coding genes			
		%A	%T	%G	%C	%A	%T	%G	%C
Arvicolinae (*Microtus fortis fortis*)	16310	32.66	26.22	13.42	27.70	30.79	27.14	13.12	28.94
Arvicolinae (*Microtus kikuchii*)	16312	32.71	25.99	13.59	27.71	30.72	27.02	13.42	28.84
Arvicolinae (*Microtus levis*)	16283	32.95	27.29	13.59	26.17	30.93	28.50	13.41	27.15
Arvicolinae (*Neodon fuscus*)	16328	31.91	26.54	14.36	27.20	30.63	26.47	13.41	29.48
Arvicolinae (*Neodon irene*)	16367	32.33	26.78	13.89	26.99	30.32	27.84	13.59	28.25
Arvicolinae (*Proedromys liangshanesis*)	16296	33.01	25.99	13.64	27.37	32.17	26.22	12.31	29.30
Cricetinae (*Mesocricetus auratus*)	16264	32.63	31.07	13.07	23.22	31.26	32.85	12.51	23.38
Murinae (*Rattus rattus*)	16305	34.01	27.89	12.57	25.53	31.72	29.32	12.37	26.59
Murinae (*Mus musculus musculus*)	16300	34.61	28.55	12.31	24.53	32.66	29.83	12.19	25.33
Sciurinae (*Scinrus vulgaris*)	16507	32.11	30.86	12.55	24.48	30.16	32.55	12.44	24.85
Leporidae (*Lepus capensis*)	17722	31.64	29.53	13.19	25.63	29.62	31.58	13.23	25.57
Ochotonidae (*Ochotona curzoniae*)	17131	31.02	25.87	13.46	29.65	28.70	27.63	13.35	30.32

### Protein-coding genes

Twelve of the 13 protein-coding genes were identified on the H-strand, while the remaining gene (*nad*6) was on the L-strand. The total length of protein-coding genes of *N. fuscus* was 11396 bp, accounting for 69.79% of the complete mt genome. As shown in Table 3, the genes encoding COX1-3, ATP6, ATP8, ND4L, ND4 and Cytb started with the common start codon ATG, whereas *nad*3 and the three remaining genes (*nad*1-2 and *nad*5) used GTG and ATA/ATT as start codons, a situation occasionally seen in other mammals. Seven genes (*cox*1, *cox*2, *atp*6, *atp*8, *nad*3, *nad*4L and *cyt*b) were terminated by the general stop codon TAA, whereas *nad*2, *nad*5 and *nad*6 used TAG as the stop codon. The remaining genes (*nad*1, *cox*3 and *nad*4) used incomplete stop codons T(–), presumably transformed into complete stop codons through post-transcriptional polyadenylation [25]. This phenomenon is widespread in other animals [26–28].

**Table 3:** Location, size, and other information of genes in the mt genome of *Neodon fuscus*

Genes	Begins	Ends	Size (bp)	Strand	Start/stop condon	Intergenic nucleotides
*tRNA^Phe^*	1	66	66	H		
12S rRNA	69	1015	947	H		2
*tRNA^Val^*	1016	1086	71	H		0
16S rRNA	1087	2648	1562	H		0
*tRNA^Leu^*	2650	2724	75	H		1
*nad*1	2722	3681	960	H	ATA/TAG	-2
*tRNA^Ile^*	3680	3747	68	H		0
*tRNA^Gln^*	3745	3816	72	L		-3
*tRNA^Met^*	3818	3886	69	H		1
*nad*2	3887	4921	1035	H	ATT/TAG	0
*tRNA^Trp^*	4923	4989	67	H		1
*tRNA^Ala^*	4991	5059	69	L		1
*tRNA^Asn^*	5062	5131	70	L		2
*O_L_*	5132	5164	33	L		0
*tRNA^Cys^*	5163	5230	68	L		-2
*tRNA^Tyr^*	5231	5297	67	L		0
*cox*1	5299	6843	1545	H	ATG/TAA	1
*tRNA^Ser^*	6841	6909	69	L		-3
*tRNA^Asp^*	6913	6980	68	H		3
*cox*2	6982	7665	684	H	ATG/TAA	1
*tRNA^Lys^*	7669	7732	64	H		3
*atp*8	7733	7936	204	H	ATG/TAA	0
*atp*6	7894	8574	681	H	ATG/TAA	57
*cox*3	8574	9357	784	H	ATG/T	-1
*tRNA^Gly^*	9358	9426	69	H		0
*nad*3	9427	9774	348	H	GTG/TAA	6
*tRNA^Arg^*	9776	9842	67	H		1
*nad*4L	9844	10140	297	H	ATG/TAA	1
*nad*4	10134	11511	1378	H	ATG/T	-7
*tRNA^His^*	11512	11579	68	H		0
*tRNA^Ser^*	11580	11638	59	H		0
*tRNA^Leu^*	11638	11707	70	H		-1
*nad*5	11708	13519	1812	H	ATA/TAG	0
*nad*6	13516	14040	525	L	ATG/TAG	-4
*tRNA^Glu^*	14041	14109	69	L		0
*Cyt*b	14115	15257	1143	H	ATG/TAA	5
*tRNA^Thr^*	15260	15327	68	H		2
*tRNA^Pro^*	15328	15395	68	L		0
D-loop	15396	16328	933	H		0

In terms of codon usage patterns, *N. fuscus* prefers to use U+A codons, as observed in most rodent mt genomes. The codon CUA was the most commonly used, accounting for 4.05% of the total codons (3832) while CGG was the least used (0.47%).

### tRNA and rRNA genes

The mt genome of *N. fuscus* had two genes for rRNAs (*12S* rRNA and *16S* rRNA) and 22 tRNA-coding genes (specifying 20 amino acids). The *12S* rRNA and *16S* rRNA genes of *N. fuscus* mtDNA were 947 and 1562 bp in length, respectively, and were separated by *tRNA^Val^*. The 22 tRNA genes (14 on the H-strand and 8 on the L-strand) ranged from 59 to 75 bp in length (Table 3). The *tRNA^Ser^* ^(GCU)^ gene lacked the dihydrouridine stem and loop (DHU), a common phenomenon among vertebrates [29,30], whereas the 21 remaining tRNA genes had typical clover-leaf structures (not shown).

### Noncoding regions

**Figure 1:**
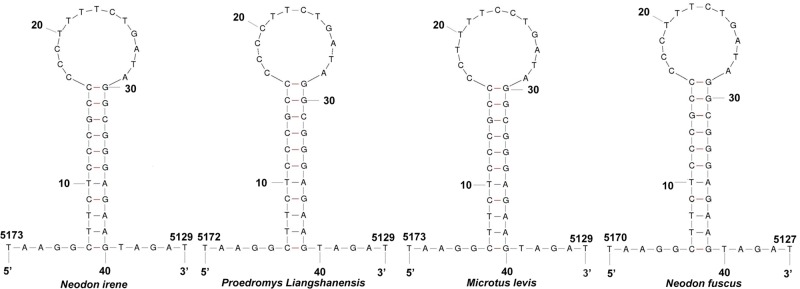
The predicted structure of the O_L_ region of *Neodon fuscus, Microtus* levis, *Proedromys liangshanensis* and *Neodon irene*

The two noncoding regions included a control region (CR) and L-strand replication origin (O_L_). The length of the CR was 933 bp, capable of forming a stable D-loop structure and was located between *tRNA^Pro^* and *tRNA^Phe^*. The D-loop region was composed of an extended termination-associated sequence (ETAS), the central domain (CD), conserved sequence blocks (CSBs), L-strand promoter and H-strand promoter. Based on the situation for other species, we could identify in the ETAS a likely hypervariable region, 251 bp in length and two conserved blocks, ETAS1 (59 bp) and ETAS2. The CD was 308 bp in length, rich in G (19.48%). Three CSBs were identifiable: CSB-1, CSB-2 and CSB3 [31,32]. CSB-1 (here, 24 bp) is the most conserved block present in most mammals, while CSB-2 only partially exists in some species and CSB-3 is sometimes absent [32,33]. The O_L_ region was 34 bp in length, extending from the 3′ end of *tRNA^Lys^* to the 5′ end of *tRNA^Asn^* within the WANCY cluster of tRNAs. It could fold into a stable stem-loop secondary structure with an 11 bp stem and a 12 bp loop (Figure 1). A comparison of the O_L_ region of four members of the Arvicolinae shows the stem region to be very well conserved while the loop region was slightly less conserved.

### Diversity of mt DNAs among vole individuals

The alignment of the *cyt*b gene and D-loop for 37 individuals of *N. fuscus*, including specimen 1, revealed that there were no differences in the *cyt*b gene, and that only two variable sites (384 and 691) existed in the D-loop sequences (Supplementary Figure S2).

### Phylogenetic analyses

**Figure 2:**
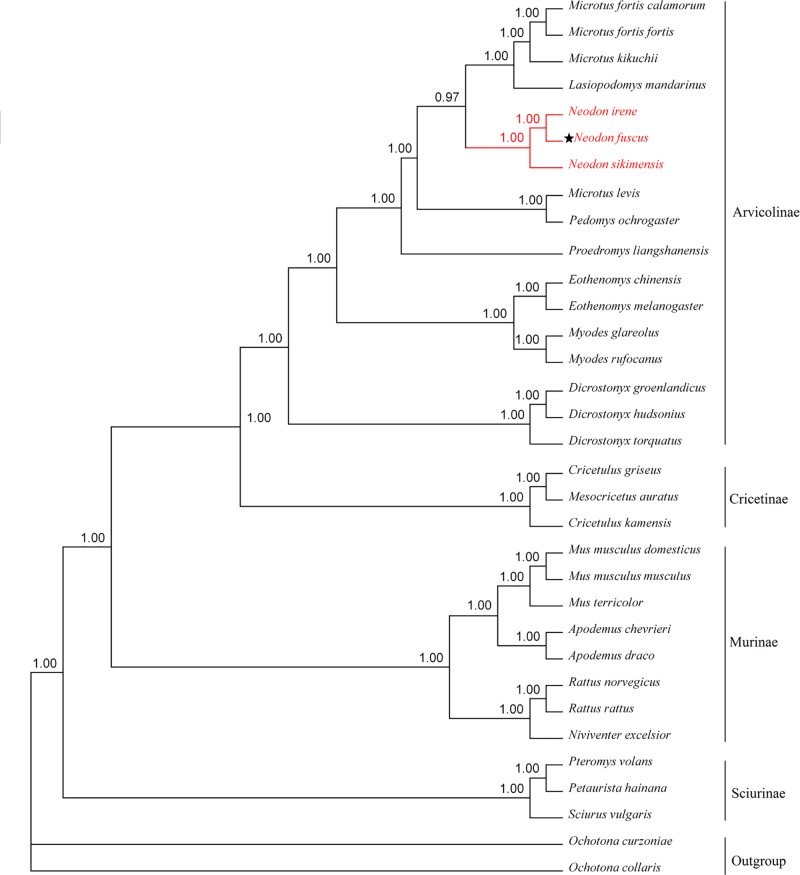
The phylogenetic relationship of *Neodon fuscus* with 32 other rodent species inferred from a Bayesian method based on an alignment of complete mt genomes The numbers at a node represent bootstrap values. ★ Indicates *N. fuscus* examined in our study. *O. collaris* and *O. curzoniae* are used as outgroups.

**Figure 3:**
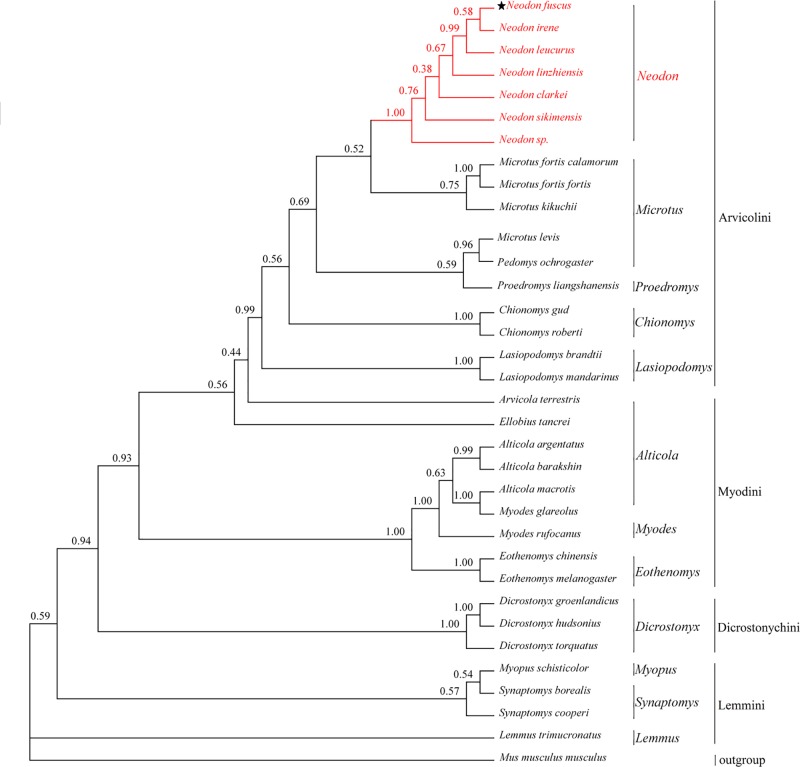
Phylogenetic trees inferred from *cyt*b gene sequences (from 34 species) using Bayesian method The number at each node represents the bootstrap value. ★ Indicates *N. fuscus* examined in our study. *Mus musculus* is used as outgroup.

Phylogenetic trees (Figure 2) demonstrate the monophyly of each subfamily or family of rodents. In both ML (not shown) and Bayesian analyses, *N. fuscus* had a closer relationship with *Neodon* species than with any member of the genus *Microtus* or *Lasiopodomys mandarinus*. Trees inferred from *cyt*b genes or an alignment of 13 concatenated protein-coding genes also grouped *N. fuscus* and the other *Neodon* species to the exclusion of other species (Figure 3; Supplementary Figure S1). In these trees, the genus *Microtus* is shown to be paraphyletic, rendered so by the position of *Microtus levis*.

## Discussion

In the present study, we determined the complete nucleotide sequence of the *N. fuscus* mt genome, which is similar to that of other rodents in gene number, arrangement, composition and other features. For instance, the content of A+T in the mt DNAs for *Microtus fortis fortis, M. kikuchii, Neodon irene, Proedromys liangshanesis* and *O. curzoniae* was under 60%, similar to that of *N. fuscus*.

In all phylogenetic analysis reported here, *N. fuscus, N. irene* and *Neodon sikimensis* were sister taxa. Similarly, the phylogenetic trees constructed by Chen et al (2012) [11] demonstrated a close relationship between *N. fuscus, N. irene* and *N. sikimensis*.

*N. irene* [34] (Irene’s mountain vole, Chinese scrub vole) occurs in alpine meadows and shrubby slopes in West China (Qinghai, Gansu, Sichuan, Xizang and Yunnan) and Myanmar, at elevations ranging from 2800 to over 4000 meters [8,35]. Its complete mt genome was sequenced recently [34,36].

Despite slight differences between IQ-TREE (ML) and Bayesian analyses, *N. fuscus* is clearly not close to *Lasiopodomys mandarinus* (*Microtus mandarinus*), which is clustered with species of *Microtus*. In other words, *N. fuscus* does not belong to genus *Microtus* and it is also inappropriate for it to be named *L. fuscus*. Liu et al [10] have suggested that *Microtus fuscus* should be transferred to *Neodon fuscus* according to morphological and molecular analysis. Our work using whole mt genomes further confirms this conclusion. Another species regularly appearing close to *N. fuscus* in phylogenetic trees was *N. leucurus*, which had previously been assigned commonly to genus *Phaiomys*. Much of their sequence data for *N. leucurus* were derived from the study by Galewski et al (2006) [37], who also found a close relationship between

*N. irene* and *N. leucurus*, but *N. fuscus* was not included in their study. Synonymy of *N*. *fuscus* with *N*. *leucurus* appears never to have been suggested. A possible explanation might be the misidentification of some of the tissues used in previous studies.

The speciose genus *Microtus* includes more than 60 species, accounting for ∼50% of Arvicoline rodents [34,38]. The apparently rapid evolution rate of *Microtus* is problematic for classification. In the past, the taxonomy of *Microtus* was mainly based on morphological differentiation, distinct karyotypes and different chromosome numbers [39,40]. However, some species have similar chromosome number and karyotype but appear morphologically different, while some are opposite [41,42]. Therefore, this genus clearly requires revision to fit phylogenetic relationships indicated by molecular data [10,11,37,43,44]. Both *Neodon* and *Proedromys* have been included within *Microtus* at various times [42,45–48] but have eventually become recognized as independent genera. Molecular systematic approaches to revision of the paraphyletic genus *Microtus*, and the Arvicolinae more broadly, are urgently needed. The molecular systematic analysis of *Microtus* based on molecular data including both mitochondrial and nuclear markers [49,50]. In the past decades, most phylogenetic studies focused on mt genomes and some species received a good distinction [41,51]. Recently, some researchers are beginning to use both mtDNA and nuclear DNA to analyze the phylogeny of genus *Microtus* and obtain good results [52–54]. In the future, the mtDNA and nuclear loci, especially the combined application of both, may play an important role in taxonomy of intricate genera.

Briefly, phylogenetic trees based on complete mtDNAs and mt *cyt*b genes for the subfamily Arvicolinae suggested that *N. fuscus*, and the other *Neodon* species are sister taxa. It is therefore appropriate that the vole, typically in the book ‘Wilson & Reeder’s Mammal Species of the World’ used as *L. fuscus*, should be called or renamed *N. fuscus*.

## Supporting information

**Supplementary Figure S1:** Phylogenetic tree inferred from the 13 concatenated protein-coding genes from 33 species using ML analysis. The number at a node represents bootstrap value. ★ Indicates *N. fuscus* examined in our study. *Ochotona collaris* and *O. curzoniae* are used as outgroups.

**Supplementary Figure S2:** The multiple sequence alignment based on the D-loop region of 37 individuals of *N. fuscus*. Highlighted are the 384^th^ and 691^th^ bases are variable.

**Supplementary Table S1:** Sequence data used in the design of primers.

**Supplementary Table S2:** Mitochondrial genomes for Glires used in this study and their GenBank accession numbers.

**Supplementary Table S3:** Codon usage in the mitochondrial genome of *N. fuscus* with each value representing the total number and percentage of codons in all the 13 protein-coding genes

## Abbreviations

CD: central domain
CR: control region
CSB: conserved sequence block
*cyt*b: cytochrome b
ETAS: extended termination-associated sequence
ML: maximum likelihood
mt: mitochondrial
PCR: polymerase chain reaction
rRNA: ribosomal RNA
tRNA: transfer RNA
