# Supplementary material for: Complete mitochondrial genomes confirm the generic placement of the plateau vole, Neodon fuscus
Source: Biosci Rep. 2019 Aug 9;39(8):BSR20182349. doi: 10.1042/BSR20182349 (PMC6689105; doi:10.1042/BSR20182349)
Supplement: Supplementary file 1 [file bsr20182349_Supp1.pdf]

Complete Mitochondrial Genomes Confirm the Generic Placement of the Plateau Vole, *Neodon fuscus*

Jian-Qiu Li[1†], Li Li[1†], Bao-Quan Fu[1], Hong-Bin Yan[1*], Wan-Zhong Jia[1, 2*]

Supporting Information

Table S1 Sequence data used in the design of primers.

Taxon	Accession number	Origin of specimen
Microtus fortis fortis	NC015241[1]	China: Ningxia
Microtus fortis calamorum	NC015243[1]	China: Hunan
Microtus kikuchii	NC003041[2]	China: Taipei
Neodon irene	NC016055[3]	China: Sichuan
Microtus levis	NC008064[4]	United States: Texas
Proedromys liangshanensis	NC013563[5]	China: Sichuan

Table S2 Mitochondrial genomes for Glires used in this study and their GenBank accession numbers.

Species	Common name	Accession number	Species Authority
Alticola argentatus	Silver Mountain Vole	DQ845186	Severtzov, 1879.
Alticola barakshin	Gobi Altai Mountain Vole	KJ556661[6]	Bannikov, 1947.
Alticola macrotis	Large-eared Mountain Vole	DQ845196	Radde, 1862.
Apodemus chevrieri	Chevrier’s field mouse	NC017599[7]	Milne-Edwards, 1868
Apodemus draco	South China field mouse	NC019584	Barrett-Hamilton, 1900
Arvicola terrestris	European water vole	AF159400[8]	Amori, 1996
Chionomys roberti	Robert’s Snow Vole	NC007936[9]	Satunin, 1903
Cricetulus griseus	Chinese hamster	NC007936	Milne-Edwards, 1867
Cricetulus kamensis	Tibetan dwarf hamster	NC024592[10]	Satunin, 1903
Dicrostonyx groenlandicus	Nearctic Collared Lemming	NC034313	Traill, 1823
Dicrostonyx hudsonius	Ungava Collared Lemming	NC034307	Pallas, 1778
Dicrostonyx torquatus	Palearctic Collared Lemming	NC034646	Pallas, 1778
Ellobius tancrei	Eastern Mole Vole	AF119270	Blasius, 1884
Eothenomys chinensis	Sichuan red-backed vole	NC013571[11]	Thomas, 1891
Eothenomys melanogaster	Père David’s Red-backed Vole	NC027418[12]	Milne-Edwards, 1871
Lasiopodomys brandtii	Brandt’s Vole	JF906120	Radde, 1861
Lasiopodomys mandarinus	Mandarin vole	NC025283[13]	Milne-Edwards, 1871
Lemmus trimucronatus	Nearctic Brown Lemming	AF119276	Richardson, 1825
Mesocricetus auratus	Golden hamster	NC013276	Waterhouse, 1839
Microtus fortis calamorum	Yangtze vole	NC015243	Thomas,1902
Microtus fortis fortis	Reed vole	NC015241	Bünchner, 1889
Microtus kikuchii	Taiwan vole	NC003041	Kuroda, 1920
Microtus levis	East European vole	NC008064	Miller, 1908
Microtus ochrogaster	Prairie Vole	NC027945	Wagner, 1842
Mus musculus domesticus	Western European house mouse	KF937873[14]	Linnaeus, 1758
Mus musculus musculus	Eastern European house mouse	NC010339[15]	Linnaeus, 1758
Mus terricolor	Earth-colored mouse	EU352649	Blyth, 1851
Myodes glareolus	Bank Vole	NC024538[16]	Schreber, 1780
Myodes rufocanus	Gray Red-backed Vole	NC029477[17]	Sundevall, 1846
Myopus schisticolor	Wood Lemming	AF119263	Lilljeborg, 1844
Neodon clarkei	Clarke’s Vole	KP190221	Hinton, 1923
Neodon irene	Irene’s mountain vole	NC016055	Thomas, 1911

Neodon juldaschi	Juniper Mountain Vole	EF599113	Severtzov, 1879
Neodon leucurus	Blyth's Mountain Vole	KP190225	Blyth, 1863
Neodon linzhiensis	unkown	HQ123618	Liu, 2012
Neodon sikimensis	Sikkim Mountain Vole	HQ123606	Horsfield, 1841
Neodon sp.1 WJ-2014	unkown	KP190217	Liu, 2014
Niviventer excelsior	Large white-bellited rat	NC019617[18]	Thomas, 1911
Ochotona collaris	Collared pika	NC003033[2]	Nelson, 1893
Ochotona curzoniae	Black-lipped pika	NC011029	Hodgson, 1858
Petaurista hainana	gaint flying squirrel	NC023089	G. Allen, 1925
Proedromys liangshanensis	Vole	NC013563	Liu, 2007
Pteromys volans	Siberian flying squirrel	NC019612[19]	Linnaeus, 1758
Rattus norvegicus	Norway rat	NC001665	Berkenhout, 1769
Rattus rattus	Black rat	NC012374[20]	Linnaeus, 1758
Sciurus vulgaris	Eurasian red squirrel	NC002369[21]	Linnaeus, 1758
Synaptomys borealis	Northern Bog Lemming	AF119259	Richardson, 1828
Synaptomys cooperi	Southern Bog Lemming	DQ323957[22]	Baird, 1857
Tscherskia triton	Greater long-tailed hamster	NC013068	de Winton, 1899

Table S3 Codon usage in the mitochondrial genome of *N. fuscus* with each value representing the total number and percentage of codons in all the 13 protein-coding genes.

Condon	Count (%)	Condon	Count (%)	Condon	Count (%)	Condon	Count (%)
UUU-Phe	60 (1.566)	UCU-Ser	68 (1.774)	UAU-Tyr	84 (2.192)	UGU-Cys	17 (0.444)
UUC-Phe	100 (2.609)	UCC-Ser	101 (2.636)	UAC-Tyr	74 (1.931)	UGC-Cys	20 (0.522)
UUA-Leu	88 (2.296)	UCA-Ser	125 (3.262)	UAA-Stop	75 (1.957)	UGA-Stop	67 (1.748)
UUG-Leu	22 (0.574)	UCG-Ser	30 (0.783)	UAG-Stop	47 (1.227)	UGG-Trp	17 (0.444)
CUU-Leu	65 (1.696)	CCU-Pro	112 (2.923)	CAU-His	77 (2.009)	CGU-Arg	15 (0.391)
CUC-Leu	84 (2.192)	CCC-Pro	85 (2.218)	CAC-His	87 (2.270)	CGC-Arg	18 (0.470)
CUA-Leu	155 (4.045)	CCA-Pro	122 (3.184)	CAA-Gln	101 (2.636)	CGA-Arg	26 (0.678)
CUG-Leu	43 (1.122)	CCG-Pro	20 (0.522)	CAG-Gln	46 (1.200)	CGG-Arg	9 (0.235)
AUU-Ile	107 (2.792）	ACU-Thr	85 (2.218)	AAU-Asn	71 (1.853)	AGU-Ser	25 (0.652)
AUC-Ile	106 (2.766）	ACC-Thr	82 (2.140)	AAC-Asn	99 (2.583)	AGC-Ser	56 (1.461)
AUA-Ile	123 (3.210）	ACA-Thr	143 (3.732)	AAA-Lys	96 (2.505)	AGA-Arg	15 (0.391)
AUG-Met	51 (1.331)	ACG-Thr	21 (0.548)	AAG-Lys	23 (0.600)	AGG-Arg	21 (0.548)
GUU-Val	24 (0.626)	GCU-Ala	46 (1.200)	GAU-Asp	33 (0.861)	GGU-Gly	19 (0.496)
GUC-Val	40 (1.044)	GCC-Ala	82 (2.140)	GAC-Asp	52 (1.357)	GGC-Gly	48 (1.253)
GUA-Val	48 (1.253)	GCA-Ala	53(1.383)	GAA-Glu	60 (1.566)	GGA-Gly	46 (1.200)
GUG-Val	24 (0.626)	GCG-Ala	15 (0.391)	GAG-Glu	27 (0.705)	GGG-Gly	30 (0.783)

Figure S1 Phylogenetic tree inferred from the 13 concatenated protein-coding genes from 33 species using ML analysis. The number at a node represents bootstrap value. ★ Indicates *N. fuscus* examined in our study. *Ochotona collaris* and *O. curzoniae* are used as outgroups.

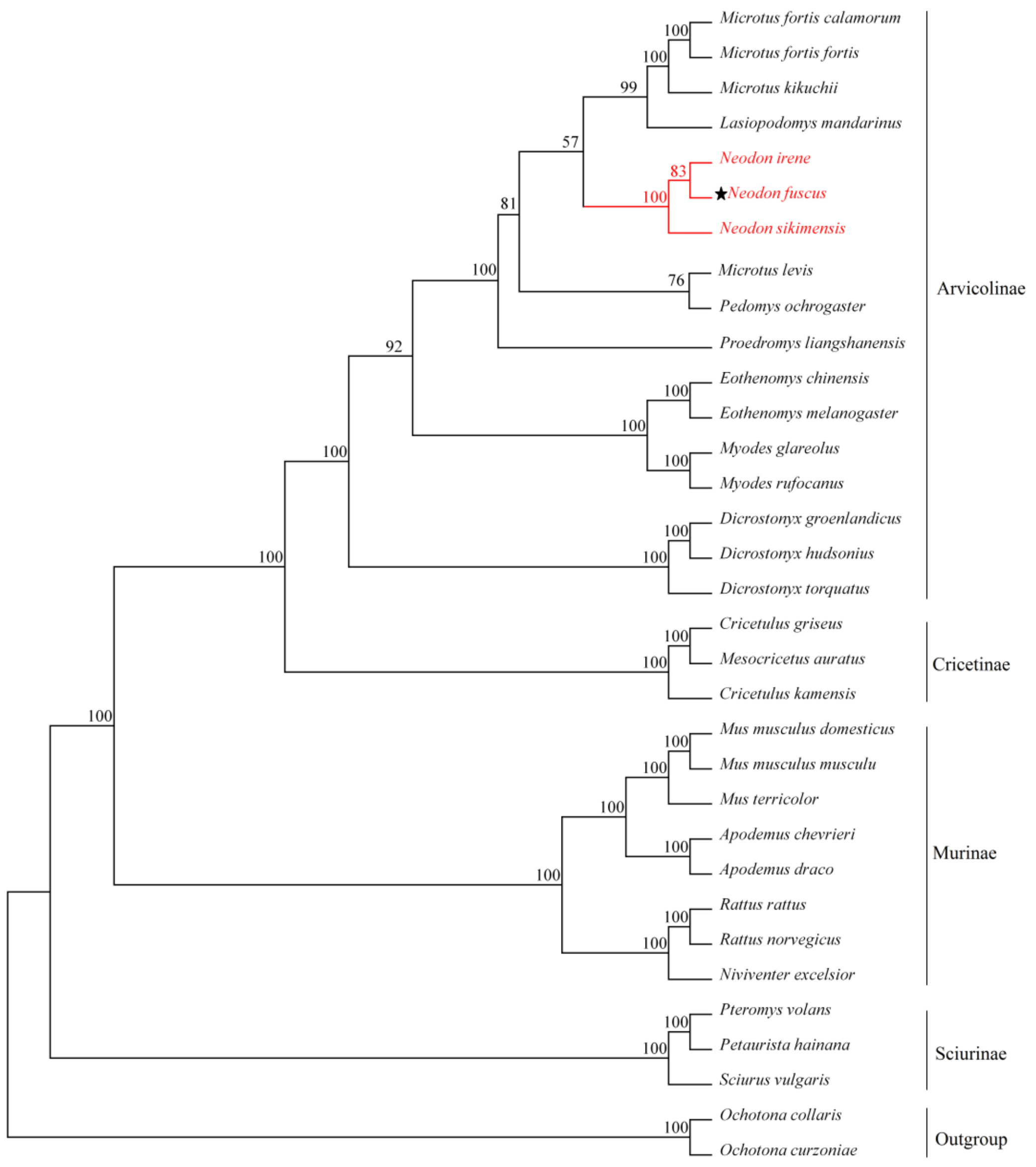

Figure S2 The multiple sequence alignment based on the D-loop region of 37 individuals of *N. fuscus*. Highlighted are the 384th and 691th bases are variable.

```
specimen1[a] 375 CAGGGCCATTCCAT-------------------------------CATACTTTTCATGCTA 700
6            375 CAGGGCCATTCCAT-------------------------------CATACTTTTCATGCTA 700
13           375 CAGGGCCATTCCAT-------------------------------CATACTTTTCATGCTA 700
14           375 CAGGGCCATTCCAT-------------------------------CATACTTTTCATGCTA 700
16           375 CAGGGCCATTCCAT-------------------------------CATACTTTTCATGCTA 700
17           375 CAGGGCCATTCCAT-------------------------------CATACTTTTCATGCTA 700
22           375 CAGGGCCATTCCAT-------------------------------CATACTTTTCATGCTA 700
29           375 CAGGGCCATTCCAT-------------------------------CATACTTTTCATGCTA 700
30           375 CAGGGCCATTCCAT-------------------------------CATACTCTTCATGCTA 700
34           375 CAGGGCCATTCCAT-------------------------------CATACTCTTCATGCTA 700
36           375 CAGGGCCATTCCAT-------------------------------CATACTCTTCATGCTA 700
33           375 CAGGGCCATTCCAT-------------------------------CATACTCTTCATGCTA 700
32           375 CAGGGCCATTCCAT-------------------------------CATACTCTTCATGCTA 700
31           375 CAGGGCCATTCCAT-------------------------------CATACTCTTCATGCTA 700
28           375 CAGGGCCATTCCAT-------------------------------CATACTCTTCATGCTA 700
27           375 CAGGGCCATTCCAT-------------------------------CATACTCTTCATGCTA 700
25           375 CAGGGCCATTCCAT-------------------------------CATACTCTTCATGCTA 700
23           375 CAGGGCCATTCCAT-------------------------------CATACTCTTCATGCTA 700
21           375 CAGGGCCATTCCAT-------------------------------CATACTCTTCATGCTA 700
18           375 CAGGGCCATTCCAT-------------------------------CATACTCTTCATGCTA 700
12           375 CAGGGCCATTCCAT-------------------------------CATACTCTTCATGCTA 700
11           375 CAGGGCCATTCCAT-------------------------------CATACTCTTCATGCTA 700
10           375 CAGGGCCATTCCAT-------------------------------CATACTCTTCATGCTA 700
9            375 CAGGGCCATTCCAT-------------------------------CATACTCTTCATGCTA 700
8            375 CAGGGCCATTCCAT-------------------------------CATACTCTTCATGCTA 700
7            375 CAGGGCCATTCCAT-------------------------------CATACTCTTCATGCTA 700
5            375 CAGGGCCATTCCAT-------------------------------CATACTCTTCATGCTA 700
4            375 CAGGGCCATTCCAT-------------------------------CATACTCTTCATGCTA 700
3            375 CAGGGCCATTCCAT-------------------------------CATACTCTTCATGCTA 700
1            375 CAGGGCCATTCCAT-------------------------------CATACTCTTCATGCTA 700
2            375 CAGGGCCATCCCAT-------------------------------CATACTCTTCATGCTA 700
20           375 CAGGGCCATCCCAT-------------------------------CATACTCTTCATGCTA 700
26           375 CAGGGCCATCCCAT-------------------------------CATACTCTTCATGCTA 700
35           375 CAGGGCCATCCCAT-------------------------------CATACTCTTCATGCTA 700
37           375 CAGGGCCATCCCAT-------------------------------CATACTCTTCATGCTA 700
15           375 CAGGGCCATCCCAT-------------------------------CATACTTTTCATGCTA 700
24           375 CAGGGCCATCCCAT-------------------------------CATACTTTTCATGCTA 700
                 ********* ****                                ****** *********
```

a: The specimen was used for sequencing of complete mtDNA in the study.

Reference:

1 Jiang, X., Gao, J., Ni, L., Hu, J., Li, K., Sun, F., Xie, J., Bo, X., Gao, C., Xiao, J., Zhou, Y. (2012). The complete mitochondrial genome of *Microtus fortis calamorum* (Arvicolinae, Rodentia) and its phylogenetic analysis. *Gene* **498**, 288-295, doi:10.1016/j.gene.2012.02.022

2 Lin, Y. H., Waddell, P. J. & Penny, D. (2002) . Pika and vole mitochondrial genomes increase support for both rodent monophyly and glires. *Gene* **294**, 119-129

3 Fan, L., Fan, Z., Yue, H., Zhang, X., Liu, Y., Sun, Z., Liu, S., Yue, B. (2011). Complete mitochondrial genome sequence of the Chinese scrub vole (*Neodon irene*). *Mitochondrial DNA* **22**, 50-52, doi:10.3109/19401736.2011.603312

4 Triant, D. A. & Dewoody, J. A. (2006). Accelerated molecular evolution in *Microtus* (Rodentia) as assessed via complete mitochondrial genome sequences. *Genetica* **128**, 95-108, doi:10.1007/s10709-005-5538-6

5 Hao, H., Liu, S., Zhang, X., Chen, W., Song, Z., Peng, H., Liu, Y., Yue, B. (2011). Complete mitochondrial genome of a new vole *Proedromys liangshanensis* (Rodentia: Cricetidae) and phylogenetic analysis with related species: are there implications for the validity of the genus Proedromys? *Mitochondrial DNA* **22**, 28-34, doi:10.3109/19401736.2011.588220

6 Kohli, B. A., Speer, K. A., Kilpatrick, C. W., Batsaikhan, N., Damdinbazar, D., Cook, J. A . (2014). Multilocus systematics and non-punctuated evolution of *Holarctic Myodini* (Rodentia: Arvicolinae). *Molecular phylogenetics and evolution* **76**, 18-29, doi:10.1016/j.ympev.2014.02.019

7 Yue, H., Fan, Z., Liu, S., Liu, Y., Song, Z., Zhang, X. (2012). A mitogenome of the Chevrier's field mouse (*Apodemus chevrieri*) and genetic variations inferred from the cytochrome b gene. *DNA and cell biology* **31**, 460-469, doi:10.1089/dna.2011.1301

8 Martin, Y., Gerlach, G., Schlotterer, C. & Meyer, A. (2000). Molecular phylogeny of European muroid rodents based on complete cytochrome b sequences. *Molecular phylogenetics and evolution* **16**, 37-47, doi:10.1006/mpev.1999.0760

9 Partridge, M. A., Davidson, M. M. & Hei, T. K. (2007). The complete nucleotide sequence of Chinese hamster (*Cricetulus griseus*) mitochondrial DNA. *DNA sequence : the journal of DNA sequencing and mapping* **18**, 341-346, doi:10.1080/10425170601101287

10 Kang, C., Yue, H., Liu, M., Huang, T., Liu, Y., Zhang, X., Yue, B., Zeng, T., Liu, S. (2016).The complete mitochondrial genome of *Cricetulus kamensis* (Rodentia: Cricetidae). *Mitochondrial DNA. Part A, DNA mapping, sequencing, and analysis* **27**, 976-977, doi:10.3109/19401736.2014.926513

11 Yang, C., Hao, H., Liu, S., Liu, Y., Yue, B., Zhang, X. (2012). Complete mitochondrial genome of the Chinese oriental vole *Eothenomys chinensis* (Rodentia: Arvicolinae). *Mitochondrial DNA* **23**, 131-133, doi:10.3109/19401736.2012.660926

12 Chen, S., Chen, G., Wei, H. & Wang, Q. (2016). Complete mitochondrial genome of the Pere David's Vole, *Eothenomys melanogaster* (Rodentia: Arvicolinae). *Mitochondrial DNA. Part A, DNA mapping, sequencing, and analysis* **27**, 2496-2497, doi:10.3109/19401736.2015.1036246

13 Cong H, Kong L, Liu Z, Wu Y, Motokawa M, Harada M, Li Y. (2016). Complete mitochondrial genome of the mandarin vole *Lasiopodomys mandarinus* (Rodentia: Cricetidae). *Mitochondrial DNA. Part A, DNA mapping, sequencing, and analysis* **27**, 760-761, doi:10.3109/19401736.2014.915528

14 Zheng, J., Chen, Y., Deng, F., Huang, R., Petersen, F., Ibrahim, S., Yu, X. (2014). mtDNA sequence, phylogeny and evolution of laboratory mice. *Mitochondrion* **17**, 126-131, doi:10.1016/j.mito.2014.07.006

15 Gregorova, S., Divina, P., Storchova, R., Trachtulec, Z., Fotopulosova, V., Svenson, K.L., Donahue,

L. R., Paigen, B., Forejt, J. (2008). Mouse consomic strains: exploiting genetic divergence between *Mus m. musculus* and *Mus m. domesticus* subspecies. *Genome research* **18**, 509-515, doi:10.1101/gr.7160508

16 Bendova, K., Markova, S., Searle, J. B. & Kotlik, P. (2016). The complete mitochondrial genome of the bank vole *Clethrionomys glareolus* (Rodentia: Arvicolinae). *Mitochondrial DNA. Part A, DNA mapping, sequencing, and analysis* **27**, 111-112, doi:10.3109/19401736.2013.873927

17 Lu, T., Zhu, M., Yi, C., Si, C., Yang, C., Chen, H. (2017).Complete mitochondrial genome of the gray red-backed vole (*Myodes rufocanus*) and a complete estimate of the phylogenetic relationships in Cricetidae. *Mitochondrial DNA. Part A, DNA mapping, sequencing, and analysis* **28**, 62-64, doi:10.3109/19401736.2015.1110799

18 Chen, W., Sun, Z., Liu, Y., Yue, B. & Liu, S. (2012). The complete mitochondrial genome of the large white-bellied rat, *Niviventer excelsior* (Rodentia: Muridae). *Mitochondrial DNA* **23**, 363-365, doi:10.3109/19401736.2012.696627.

19 Ryu, S. H., Kwak, M. J. & Hwang, U. W. (2013). Complete mitochondrial genome of the Eurasian flying squirrel *Pteromys volans* (Sciuromorpha, Sciuridae) and revision of rodent phylogeny. *Molecular biology reports* **40**, 1917-1926, doi:10.1007/s11033-012-2248-x

20 Robins, J.H., McLenachan, P.A., Phillips, M.J., Craig, L., Ross, H.A., Matisoo-Smith, E. (2008). Dating of divergences within the Rattus genus phylogeny using whole mitochondrial genomes. *Molecular phylogenetics and evolution* **49**, 460-466, doi:10.1016/j.ympev.2008.08.001

21 Reyes, A., Gissi, C., Pesole, G., Catzeflis, F. M. & Saccone, C. (2000). Where do rodents fit? Evidence from the complete mitochondrial genome of Sciurus vulgaris. *Molecular biology and evolution* **17**, 979-983, doi:10.1093/oxfordjournals.molbev.a026379

22 Triant, D. A. & Dewoody, J. A. (2007). Extensive mitochondrial DNA transfer in a rapidly evolving rodent has been mediated by independent insertion events and by duplications. *Gene* **401**, 61-70, doi:10.1016/j.gene.2007.07.003.
